# Salivary proteomic profile of dogs with and without dental calculus

**DOI:** 10.1186/s12917-020-02514-0

**Published:** 2020-08-19

**Authors:** Mayara Bringel, Paula Karine Jorge, Priscila Amanda Francisco, Cadance Lowe, Robinson Sabino-Silva, Bella Luna Colombini-Ishikiriama, Maria Aparecida de Andrade Moreira Machado, Walter Luiz Siqueira

**Affiliations:** 1 College of Dentistry, University of Saskatchewan, Saskatoon, SK Canada; 2 Department of Pediatric Dentistry, Bauru School of Dentistry - University of São Paulo, Bauru, SP Brazil; 3 College of Veterinary Medicine, University of Saskatchewan, Saskatoon, SK Canada; 4 Department of Physiology, Institute of Biomedical Sciences, Federal University of Uberlandia, Uberlandia, Minas Gerais Brazil

**Keywords:** Mass spectrometry, Saliva, Dogs, Proteome, Dental Calculus

## Abstract

**Background:**

Dogs’ saliva is a complex mixture of inorganic and organic constituents, rich in proteins. Therefore, knowing the saliva composition of these animals is extremely important to identify the presence of proteins that may be involved in physiological and pathological mechanisms of their oral cavity. The present study aimed to characterize the proteomic profile of saliva from dogs with and without dental calculus.

**Results:**

Saliva samples were collected from 20 dogs. Before the collection, a visual clinical examination was performed and 8 subjects (40%) did not present any signs of dental calculus, while 12 (60%) presented dental calculus. After saliva collection, the samples were submitted to protein quantification (mBCA), and then they were prepared for analysis by nLC-ESI-MS/MS. A total of 658 unique proteins were identified, of which 225 were specific to dogs without dental calculus, 300 were specific to dogs with dental calculus, and 133 were common to all subjects. These proteins presented functions including transportation, immune response, structural, enzymatic regulation, signal transduction, transcription, metabolism, and some proteins perform functions as yet unknown. Several salivary proteins in dogs with dental calculus differed from those found in the group without dental calculus. Among the abundant proteins detected in periodontal affected cases, can be highlighting calcium-sensing receptor and transforming growth factor beta. Enrichment analysis reveled the presence of Rho GTPases signaling pathway.

**Conclusions:**

This research identified salivary proteins, that should be further investigated as potencial biomarkers of chronic periodontits with dental calculus formation in dogs.

## Background

Saliva has a complex mixture of organic and inorganic constituents. The organic constituents are predominantly salivary proteins [1]. Its proteome is represented especially by glycoprotein, enzymes, immunoglobulins, and several peptides [2]. Moreover, the whole saliva is composed of gingival crevicular fluid (containing plasma proteins), food debris and substances produced by oral microorganisms [3, 4].

In mammals, the main saliva functions are lubrication and protection of the buccal tissue, buffer capacity, maintenance of teeth integrity, and antimicrobial effect [3]. In some species, some saliva components represent an essential part on the enzymatic digestion assisting mastication and deglutition [5]. Besides these beneficiais functions, dogs’ saliva has an alkaline pH that varies between 7.2 to 8.5 [6, 7], a range that facilitates dental calculus formation, through calcification of dental biofilm present on teeth cervical region [8–10]. Dental calculus formation is always preceded by the development of a saliva protein layer on the tooth enamel’s surface, called acquired enamel pellicle (AEP), which serves both to protect dental enamel, reduce friction between teeth and oral mucosa [11], and as basis to formation and maturation of dental biofilm, which constitutes the organic matrix to its possible calcification [12].

In dogs, the formation of dental calculus is typical since the first year of life and it appears as granular, yellow–brown masses on the buccal surfaces of molar teeth of the upper jaw near salivary duct orifices [7, 13]. Dental calculus consists in a mixture of calcium carbonate and calcium phosphate, presenting a rough surface that increases the occurrence of periodontal disease [8], due to the calcification of more dental biofilm, bringing it closer to the soft tissues [14]. Similar to humans, there is a link in dogs between the calculus presence with the increasing rate of periodontal diseases, which in some cases may lead to the animal starvation as a result of feeding difficulties [13, 15, 16]. Furthermore, in dogs, dental calculus is considered one of the main conditions involved in the development of periodontal disease resulting in teeth loss. Studies report prevalence of periodontal disease varying from 80 to 85% [10, 17–19].

Other than constituents involved in the formation of dental calculus, saliva possesses chemical and physical properties that may serve to detect evidence, through proteomic analysis, of systemic diseases [2]. Mass spectrometry is an indicated method for studies focused on protein identification, characterization and quantification [20]. This analysis is available both for humans [21, 22] and animals [23–25]. Some proteins are involved in the formation and in the inhibition of minerals precipitation over human dental surface, such as cystatins, statherin and acidic proline-rich proteins (PRPs), which have been already identified in saliva protein composition of some mammal species like monkeys, rats, mice’s and pigs [23]. However, literature is still limited concerning identification of these proteins in dogs’ saliva. Studies with a proteomic approach may be used to develop biological strategies for controlling both bacterial dental biofilm and dental calculus formation. Thus, the purpose of this study was to characterize the salivary proteomic profile of dogs with and without dental calculus.

## Results

### Characterization of the study participants

Of the 20 dogs that participated in the study, the visual clinical examination showed that 8 (40%) had no signs of dental calculus and 12 (60%) presented with some degree of dental calculus (Table 1). The distribution of these dogs according to breed, gender, age, weight, diet and volume of saliva collected is also shown in Table 1.

**Table 1:** Distribution of the participating dogs by breed, gender, age, weight, diet and volume of saliva collected

	Shih tzu	Lhasa apso	Gender	Age (years)	Weight (Kg)	Diet^a^	Volume of saliva (μl)	Degree of dental calculus	Total Protein concentration (ug/ml)
Dog 1	X		Female	6	4	Mixed	450	0	2453.49
Dog 2		X	Female	9	8	Dry	400	3	742.24
Dog 3		X	Male	5	6.5	Dry	500	2	520.76
Dog 4		X	Male	3	6	Dry	500	2	441.77
Dog 5	X		Female	0.42	4	Dry	500	0	616.30
Dog 6	X		Female	2	5.5	Dry	400	1	546.82
Dog 7		X	Female	6	8	Dry	550	0	485.16
Dog 8		X	Female	4	8	Dry	550	1	968.05
Dog 9		X	Female	1	3	Dry	400	0	1151.45
Dog 10		X	Female	4	11	Dry	800	0	1212.73
Dog 11		X	Male	0.25	4	Dry	400	0	3171.34
Dog 12		X	Male	9	10	Dry	550	3	1972.65
Dog 13	X		Male	2	3	Dry	500	1	2168.71
Dog 14	X		Female	3	4.5	Dry	500	1	1155.82
Dog 15	X		Female	7	4	Dry	100	1	1995.13
Dog 16	X		Male	4	6.7	Mixed	400	3	1820.04
Dog 17	X		Male	0.66	7	Dry	1000	0	1633.00
Dog 18		X	Female	7	8	Mixed	600	1	2302.20
Dog 19	X		Female	0.25	2.4	Dry	700	0	1020.11
Dog 20		X	Female	7	5	Food	500	3	2360.05

N/A (Not available)

^a^A mixed diet refers to dry dog food and human food

### Total protein concentration

The concentrations of total proteins present by the Micro Bicinchoninic Acid (Micro BCA) assay in each dog studied are shown in Table 1. Student’s t-test was used to compare total protein concentrations between the groups with and without dental calculus and between genders. There were no statistically significant differences (*p* = 0.892) between groups and gender (*p* = 0.3822).

### Correlations between variables

Pearson’s correlation test (*p* < 0.05) was used to investigate the associations between animal age, saliva volume and total protein in the saliva. As expected, there was no statistically significant correlation between the variables.

### Proteome identification and quantification

**Fig. 1:**
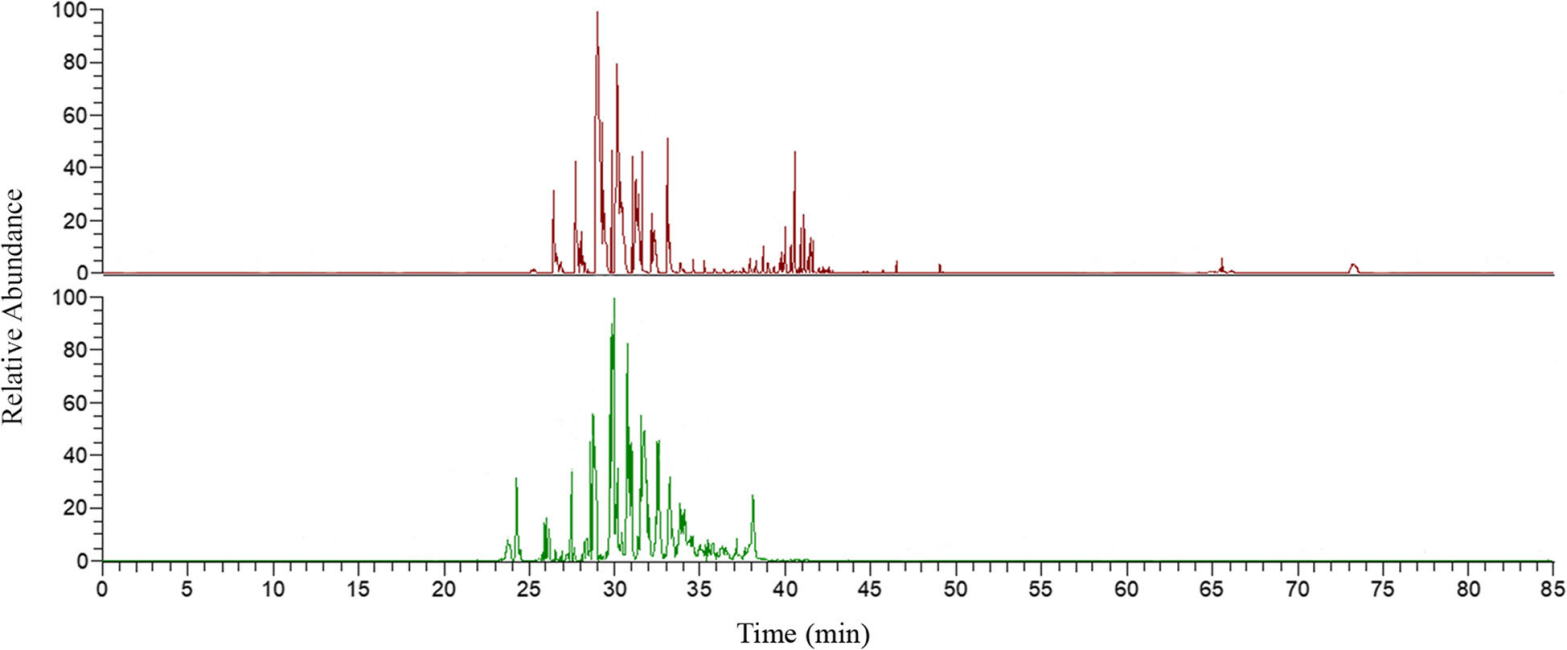
Examples of base-peak chromatograms from samples of dogs without dental calculus (upper graph) and dogs with dental calculus (lower graph) performed by nano-flow RP-HPLC column, and elution gradient ranging from 0 to 80%

The proteome from all different samples showed a consistent elution of protein/peptides range from 20 to 45 min. Representative base-peak chromatograms of saliva from dogs with and without dental calculus are represented in Fig. 1.

In the present study, 1662 proteins were identified, of which 658 (39.6%) were unique proteins. Of these 658 unique proteins found, 623 (9.7%) were characterized and 35 (15.3%) were uncharacterized proteins, therefore identified for the first time.

**Fig. 2:**
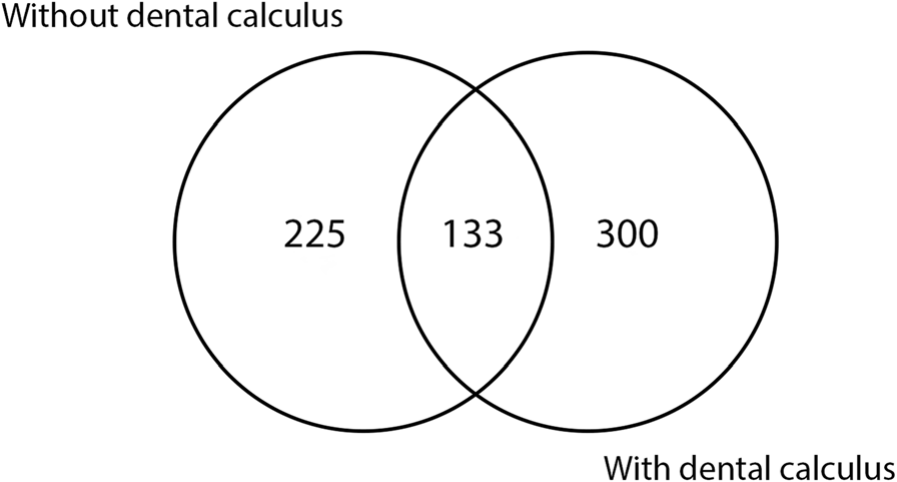
Venn diagram showing salivary proteins found in animals with and without dental calculus and their interrelationship

Regarding the oral clinical condition of each animal, 133 (20.2%) specific proteins were common to all participating dogs (Supplementary Table S1), 225 (34.2%) specific proteins were identified in dogs without clinical signs of dental calculus (Supplementary Table S2), 300 (45.6%) specific proteins in dogs with dental calculus (Supplementary Table S3). This distribution is described in Venn diagram (Fig. 2). Of these 225 specific proteins of dogs without dental calculus, 220 (97.8%) were characterized and 5 (2.2%) were uncharacterized proteins. From the 300 specific proteins of dogs with dental calculus, 287 (95.7%) present characterization and 13 (4.3%) were uncharacterized proteins.

### Biological functions of proteins

Among the 658 unique proteins identified, the most abundant based on spectral count and ion abundance [26] had their biological functions analyzed by UniProt software (www.uniprot.org). These proteins exhibited functions of substance transport, immune response, enzymatic regulation and metabolism (Table 2 and Supplementary Table S4 with peptide sequences).

**Table 2:** Functions of the most abundant proteins identified in all participants dogs

Function	Accession number	Protein name (Gene name)
Transport	F2Z4Q6	Serum albumin (AFP ALB)
	P49822	Albumin (allergen Can f 3) (ALB)
	P60524	Hemoglobin subunit beta (HBB)
	J9P430	Transferrin (TF)
	P02648	Apolipoprotein A-I (APOA1)
	P60529	Hemoglobin subunit alpha (HBA)
Immune response	J9P732	EF-hand domain-containing protein (S100A9)
	E2RCC8	Uncharacterized protein
	C0LQL0	Protein S100 (S100A8 or S100A6)
	F1PCH3	Enolase 1 (ENO1)
	P19006	Haptoglobin (HP)
	F1PR54	Uncharacterized protein
Enzymatic regulation	E2R0H6	Prolactin induced protein (PIP)
	F1PGM1	Complement C3 (C3)
	F6USN4	Uncharacterized protein
Metabolism	F1PE28	Transketolase (TKT)

Regarding the 225 specific proteins identified in dogs without dental calculus, the most abundant were: Can f 4 variant allergen, Keratin, type II cytoskeletal 1 and others. These proteins also exhibited functions of transport, immune response, transcription, structural, enzymatic regulation, metabolism and signal transduction (Table 3 and Supplementary Table S4 with peptide sequences).

**Table 3:** Functions of the most abundant specific proteins identified in dogs without dental calculus

Functions	Accession number	Protein name (Gene name)
Transport	J9P950	Can f 4 variant allergen (OBP)
	E2QX44	Solute carrier family 29 member 4 (SLC29A4)
	E2RSV0	Importin 4 (IPO4)
Immune response	E2RF74	Sphingosine-1-phosphate receptor 3 (S1PR3)
	F1PIL9	Interleukin 1 receptor accessory protein (IL1RAP)
Transcription	F1PII4	Uncharacterized protein
	E2RR25	BTB domain containing 8 (BTBD8)
Structural	F1PKA4	MTSS I-BAR domain containing 1 (MTSS1)
	F1PTY1	Keratin 3 (KRT3)
Enzymatic regulation	E2RKG0	Dermokine (DMKN)
Metabolism	E2QW50	Zinc finger protein 532 (ZNF532)
	E2RTI2	Chromodomain helicase DNA binding protein 3 (CHD3)
	F6Y4F1	Dachsous cadherin-related 1 (DCHS1)
	F1PBU5	SMG1 nonsense mediated mRNA decay associated PI3K related kinase (SMG1)
	F1Q0K5	Triokinase and FMN cyclase (TKFC)
Signal transduction	F1PSR2	Dedicator of cytokinesis 5 (DOCK5)

And finally, from the 300 specific proteins identified in dogs with dental calculus, the most abundant analyzed were: Calcium-sensing receptor, Voltage-dependent T-type calcium channel subunit alpha, Multidrug and toxin extrusion protein (Fragment), Phosphatase and actin regulator, Poly [ADP-ribose] polymerase, Zinc alpha-2-glycoprotein 1 (Fragment), Transforming growth factor beta (TGFB1) and others. These proteins exhibited functions of transport, immune response, enzymatic regulation, signal transduction, transcription and metabolism (Table 4 and Supplementary Table S4 with peptide sequences).

**Table 4:** Functions of the most abundant specific proteins identified in dogs with dental calculus

Functions	Accession number	Protein name (Gene name)
Transport	A2SXS6	Calcium-sensing receptor (CASR)
	E2R9S8	Voltage-dependent T-type calcium channel subunit alpha (CACNA1I)
	E2RAB8	Multidrug and toxin extrusion protein (SLC47A2)
Immune response	F1PI70	Transforming growth factor beta (TGFB1)
	E2R141	Complement C8 beta chain (C8B)
	F1PSJ1	Uncharacterized protein
Enzymatic regulation	E2RGH9	HECT domain E3 ubiquitin protein ligase 1(HECTD1)
	F1PVE1	PH domain and leucine rich repeat protein phosphatase 1 (PHLPP1)
	F1P6R1	Phosphatase and actin regulator (PHACTR4)
Metabolism	E2R0T6	Heat shock protein family A (Hsp70) ember 8 (HSPA8)
	E2RF62	Unc-51 like autophagy activating kinase 2 (ULK2)
	E2RD14	Pleckstrin homology domain containing A5 (PLEKHA5)
	J9P7C9	Poly [ADP-ribose] polymerase (PARP14)
Signal transduction	E2RSB9	Misato mitochondrial distribution and morphology regulator 1 (MSTO1)
Transcription	F1PQQ7	Nuclear receptor corepressor 2 (NCOR2)
Uncharacterized	F1PPE5	Biorientation of chromosomes in cell division 1 like 1 (BOD1L1)
	Q4GX49	Zinc alpha-2-glycoprotein 1(AZGP1)
	F1PI98	NCK associated protein 5 (NCKAP5)
	F1P9Y0	NHS like 1 (NHSL1)
	J9P0U6	NHS actin remodeling regulator (NHS)

**Fig. 3:**
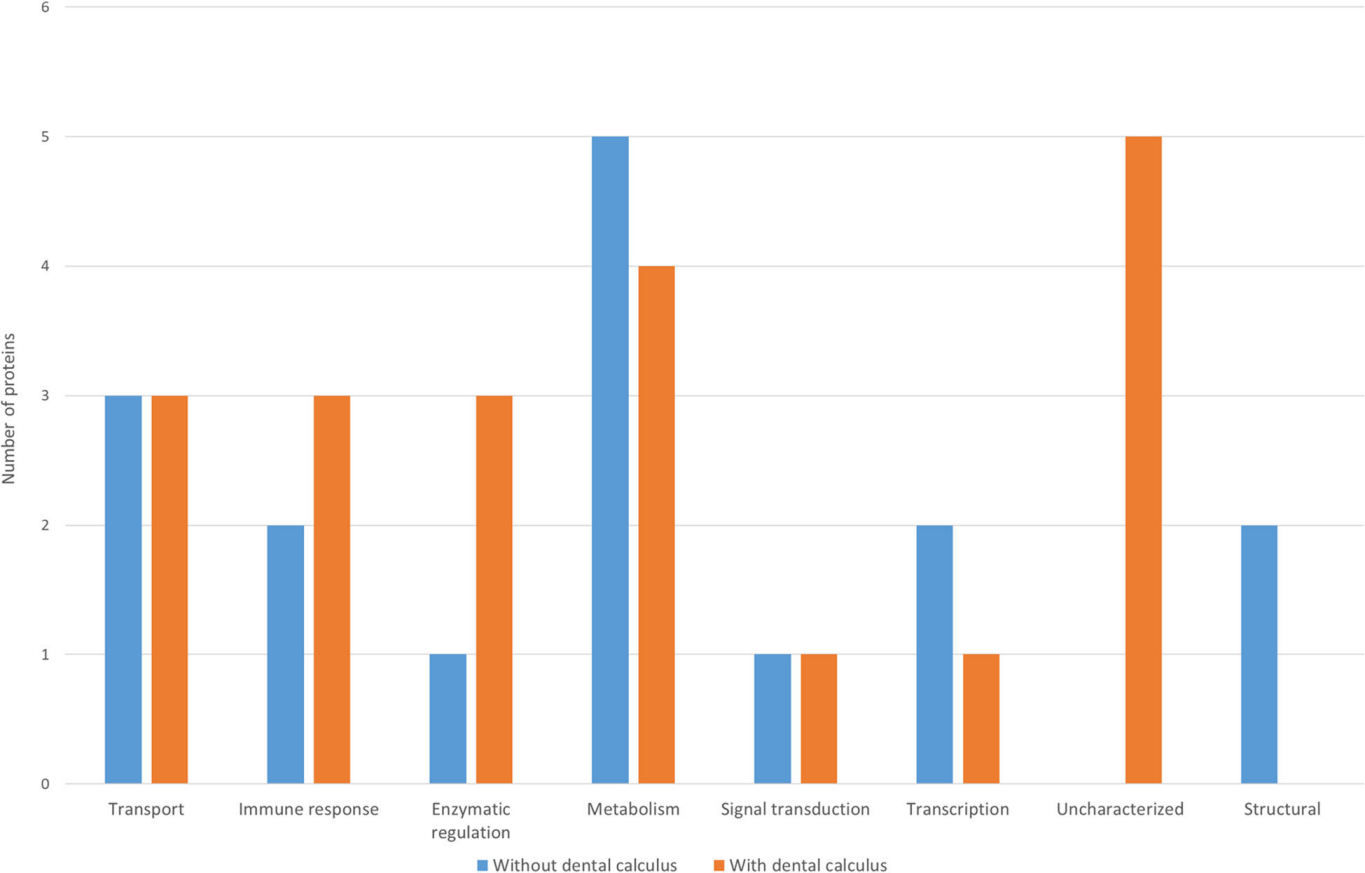
Comparison of functional characteristic of the most abundant proteins found in dogs with and without dental calculus

Fig. 3 shows and compares the functions of the most abundant proteins for dogs with and without dental calculus. The group with dental calculus presented with more proteins related to immune response, enzymatic regulation and uncharacterized proteins, as compared to the group without dental calculus.

### Analysis by the STRING database

Two protein-protein networks were created with the 225 unique proteins identified in the group of dogs without dental calculus, and 300 unique proteins in the group of dogs with dental calculus. *Canis lupus* was selected as the studied organism and the highest score of confidence (0.900) was set. Supplementary Figures 1 and 2 shows the constituents of both networks, dogs without dental calculus and dogs with dental calculus, respectively. Although it is possible to see an increase of connections from the group without dental calculus (42 edges) to the group with dental calculus (90 edges), only Reactome pathways such as cell cycle, mitosis, signaling by Rho GTPases, M phase and mitotic prometaphase were found in the second group (Supplementary Table S5). No pathways (from Reactome Pathway Datasabe or Kyoto Encyclopedia of Genes and Genomes) were encountered in the first group.

## Discussion

The current study characterized the salivary proteome profile of dogs with and without dental calculus. In total, we identified 1662 proteins through the SEQUEST filter criteria applied to MS/MS spectra. Among these salivary proteins, there were 658 (39.6%) described for the first time in saliva of dogs. Besides, it is important enphazises that 225 specific proteins were identified in dogs without clinical signs of dental calculus and 300 specific proteins of dogs with dental calculus, which demonstrated that saliva could be a valuable medium to get biomarkers of the dental calculus formation on dogs.

The whole saliva collection was performed by mechanical stimulation with aid of a device formulated for such purpose. The device used was Micro•SAL™ saliva collection device (Oasis Diagnostics® Corporation - Vancouver, WA, USA). The saliva collection device has no cellulose in its composition [27] and allows immediate visual confirmation of the volume of saliva obtained; the plunger compresses the absorbent pad and the saliva is collected in the Eppendorf tube portion of the device. Foremost, differently from previous studies where the collection of saliva in dogs was performed under anesthesia with stimulation with acid [28, 29], we have opted for a non-invasive collection without any other stimulation mechanism or general anesthesia. However, there were difficulties regarding the sample collection. The sample volumes obrained varied significantly due to the dog’s defense movements, such as head shaking or trying to move away from the owner’s containment position. These reports were also described by dog’s owners in the study of Wenger-Riggenbach et al. [30]. Moreover, some dogs refused to open their mouths and became aggressive, consequently resulting in them being excluded from the study.

The visual clinical examination of the 20 analyzed dogs identified that 8 (40%) did not present any signs of dental calculus, and 12 (60%) presented some degree of dental calculus. The mean dental calculus scores of dogs with dental calculus was 1.8, indicating a moderate calculus scoring in these dogs. Since our main goal is to study dental calculus, we selected the breeds Shih Tzu and Lhasa Apso that possesses brachycephalic skulls and tend to show malocclusion, dental crowding and rotation [31, 32], which facilitates accumulation of dental calculus. It is known that dental calculus is a predisposing factor to periodontal disease, which is more frequent in middle-aged (above 7 years old) and small size animals (below 10 kg) [33]. In addition, the breeds studied here are similar in appearance and the genetic investigation presented a close relationship between them [34]. As our study only sampled these genetically similar breeds, this may have influenced the number of proteins identified. Sousa-Pereira et al. [25] identified 249 proteins on a mixed breed group and Torres et al. [35] identified 2.491 proteins in healthy dogs among 19 breeds. Notwithstanding, there is no description of oral health on the animals evaluated in the study of Sousa-Pereira et al. [25] nor the proteins functions. In this present study were identified 1.662 proteins, of which 658 (39.6%) were unique. The biological functions of the most abundant proteins of each group identified were: transportation, immune response, structural, enzymatic regulation and metabolism, and some proteins perform still unknown functions. Besides that, in our study, there was no difference in total protein concentration between males and females. The same result was found in the study by Lucena et al. [29], in which there was no major differences between genders.

In the last years, several studies have been carried out on proteomic analysis in different mammals. Sousa-Pereira et al. [25], analyzed the salivary proteome of distinct species, including dogs, and identified, the alpha amylase (among other) was not found in the present study. There are, differences between that study and the present one that make a direct comparison of results difficult. In Sousa Pereira study [23], the samples were collected in mixed breed while in our study two specific breeds were selected. Sanguansermsri et al. compared the salivary proteome between dogs and humans, and observed that alpha amylase was present in dogs saliva, but in lower levels than in humans saliva; however, the presence of this protein in dogs in that study may be due the fact that in Thailand these animals are often fed with rice and starch [35]. Most likely due to a diet adaption, the dogs used in this study do not have this protein. This lack of amylase in dog’s saliva was also observed by other studies [4, 36, 37].

To the best of our knowledge, there are no proteomic studies regarding dental calculus in the saliva of dogs, so this is a pioneering study. Among the unique proteins identified, the existence of serum albumin was noted on every dog’s sample due to, as described in humans, a small volume of gingival crevicular fluid that enters the oral cavity even in healthy subjects, free of gingivitis, resulting the measurement of levels of this component of serum in saliva [38]. Other components of serum have also been identified, among these elements, are: Hemoglobin subunit beta, Apolipoprotein A-I, Hemoglobin subunit alpha and Haptoglobin.

Further, mucin and lysozyme C proteins were identified. Mucins are glycoproteins of high molecular weight with elongated structure that contribute significantly to saliva’s viscoelastic behavior [39]. They also play an antibacterial function of modulating selectively the coherence of microorganisms to the oral tissue surface, which assist with the control of bacterial and fungal colonization [3]. Lysozymes, on the other hand, are enzymes with hydrolytic activities. In other words, they promote cell lysis in bacteria [40], since its biochemically function hydrolyze beta-1,4 bond between N-acetyl glucosamine and N-acetyl muramic acid residues of bacterial peptidoglycan, which is an essential part of bacterial cell wall and also promotes structural stiffness and neutralize osmotic pressure of the cytoplasm [41]. Therefore, the lysozyme is considered a natural antibiotic and an important part of the innate immune system [41–43]. In humans, C-type lysozyme is found in all biofluids, including saliva. The C-type lysozymes, as the ones identified, are the main lysozymes produced by vertebrates [44].

Concerning the analyses in the two different groups, the most abundant proteins in the group of dogs without dental calculus participated in metabolism functions, whereas in the group with dental calculus there were major proteins related to immune response, enzymatic regulation and uncharacterized proteins, potentially this may be due the presence of dental calculus near the gingiva, which has tendency of more pronounced inflammatory response [45].

Among the specific proteins of dogs without dental calculus, the presence of Sphingosine-1-Phosphate Receptor Protein 1 (S1P1) was observed. This protein is highly expressed in humans in endothelial cells, brain, heart and immune system cells [46, 47]; it is coupled to G protein and binds to sphingosine-1-phosphate, which is a bioactive sphingolipid that behaves as an intracellular messenger of some cytokines and also as an autocrine and paracrine extracellular mediator [48]. In addition, sphingosine-1-phosphate stimulates events of intracellular signaling, such as activation of phospholipase C, increased cytoplasmic calcium concentration, regulation of adenylate cyclase, activation of the MAP kinase pathway and the Rho cascade [49]. When bound, they participate in several cellular processes such as proliferation, differentiation, adhesion, motility, angiogenesis, apoptosis, migration, morphogenesis and changes in the cytoskeleton [50]. S1P1 proteins were also described in memory T cells and a cell immunophenotyping revealed that humans secrete CD4(+) T cells in saliva [51]. Besides, the Voltage-dependent T-type calcium channel subunit alpha protein encoded by CACNA1G gene was equally identified as a specific protein of dogs without dental calculus. Interestingly, a similar protein encoded by CACNA1I gene was only identified in the saliva of dogs with dental calculus. It is deemed that proteins T-type calcium channel play important roles in neuronal activity and have been described in studies with rats [52] and humans [53]; but there are no reports in the literature relating these proteins to the dogs’ saliva.

According to Zhang and collaborators [54] up to 40 proteins can be named as protein biomarkers for periodontal diseases in humans, these biological mediators are released from host defense cells due to the presence of periodontopathic bacteria in the oral enviromment. They include numerous cytokines, such as prostaglandin E2; tumor necrosis factor (TNF); interleukins IL-1 and IL-6, proteinases as matrixmetalloproteinases (MMPs); elastase-like enzymes; trypsin-like proteases; aminopeptidases and dipeptidylpeptidases, epidermal; platelet-derived and vascular growth factors, pyridino-line cross-linked carboxyterminal telopeptide, osteocalcin, among others. Still, there is a lack of proteomic researches regarding dogs with chronic periodontitis, which compromises comparisons within this species.

Howsoever, amidst the specific proteins identified in dogs with dental calculus in this study, some were found to function as immune response to the current peridodontitis. Between then it can be pointed out the presence of fragments of the Polymeric immunoglobulin receptor protein, which has been described in human saliva having the function of transporter of IgA, the main salivary antibody [39]. Transforming growth factor beta (TGFB1) and complement C8 beta chain (C8B), were equally present in high abundance. TGFB1 was previously correlated to chronic periodontitis [55], its importance lies in the fact that this growth factor stimulate tissue remodeling and wound healing through increasing fibroblast proliferation, angiogenesis, and extracellular matrix production [56], and by inhibiting MMPs [57]. Paradoxically, TGFB1 was also found to promote inflammation-associated tissue degradation by enhanced production of mediators that raise recruitment of eosinophils, lympocytes and monocytes, cells known to participate in chronic inflammation and tissue destruction [58]. These facts could explain why TGFB1 is upregulated in cases of chronic periodontal pathologie.

Furthermore, another abundant protein detected by this research was the Calcium-sensing receptor (CaSR), whose function is calcium (Ca^2+^) transportation and regulation [54]. In humans, CaSR is a G protein-coupled receptor that detects extracellular levels of Ca^2+^, which is expressed on plasma membranes of a broad variety of epithelial tissues including parathyroid, kidney, gastrointestinal tract and salivary glands [59, 60]. In salivary glands, it was showed that functional CaSR proteins can be stimulated by Ca^2+^ concentration, that is, CaSR can serve as a Ca^2+^ sensor in the luminal membrane of salivary gland ducts and regulate reabsorption of Ca^2+^ from the saliva via transient receptor potential canonical 3 (TRPC3), thus contributing to maintenance of salivary Ca^2+^ levels and representing a possible important protective mechanism against formation of salivary gland stones [60]. Hence, as the CaSR stimulation in salivary glands can be related to an imbalance in the salivary Ca^2+^ concentration, it could as well contribute to the dental calculus accumulation that consist essentially of calcium phosphate. It is also important to note that in the kidney, the formation of stones is associated with changes in calcium reabsorption, causing hypercalcemia [59]. Remarkably in the current investigation, CaSR was identified only in the saliva of dogs with dental calculus, which could suggest, together with its high abundance, an association with dental calculi presence. Further research is needed to better understand the relevance of CaSR protein in saliva and dental calculi formation, as it was demonstrated mainly in other human tissues [59].

An additional layer of information on possible pathways and processes involved in periodontal disease was granted by enrichment analysis. Overall, four of the five biological process that were encountered in dental calculus cases related to mitotic pathways that is the most common eukaryotic cell cycle. However, one critical finding merit being highlighted, which is the presence of the Rho GTPases signaling pathway. Rho GTPases can modulate the effects on human periodontal ligament cells of TNFB1, an important cytokine already known by its role in periodontal pathologies [61]. Rho is a notable coordinator of the cytoskeleton [62], it was suggested in a previous study that the small Rho GTPase and its downstream effector Rho kinase (ROCK) regulate TGFB1-induced remodelling of mammary epithelial cell-to-cell contact [63]. In accordance to the stated, Wang et al. showed that TGFB1 can induce proliferation and cytoskeletal rearrangement in periodontal ligament cells via Rho GTPase-dependent pathways [64]. The high abundance of TGFB1 in dogs with dental calculus associated with the presence of Rho GTPase pathway is a relevant finding that may suggest, for futher investigations, TGFB1 as a biomarker candidate of periodontal disease in this species.

Future studies are needed for the evaluation of parameters such as pH values and determination of the buffer activity of each saliva sample collected. Quantification of electrolytes such as calcium and phosphate, which participate in the formation of the dental calculus and processes of demineralization and remineralization of dental enamel, should also be performed, not forgetting to mention sodium, potassium, zinc and magnesium, that are important in the metabolism of the salivary glands [65, 66]. The final task would be to combine the genomic, proteomic and other omic profiles together in an attempt to obtain a broader vision of how dental calculus accumulation impact dog salivary proteomic profile.

## Conclusion

In this study, the proteome salivary profile of dogs showed a large number of unique proteins, 225 belonging to dogs without dental calculus, 300 exclusives to dogs with dental calculus and 133 belonging to both groups. Therefore, this research identified salivary proteins, that should be further investigated as potencial biomarkers of chronic periodontits with formation of dental calculus in dogs. Besides, it could open opportunities to study and potentially develop new substances, that would aid in preventing or delaying the formation of dental calculus in dogs.

## Methods

### Sample selection

This study was approved by the Ethics Committee in the Teaching and Research on Animals of the Dental School of Bauru, University of São Paulo (number 002/2016); moreover, all the dogs’owners signed consent forms.

The study was composed by 20 dogs (*Canis lupus familiaris*) ranging in age from three months to nine years with and without dental calculus. General inclusion criteria of the groups with and without dental calculus included: dog breed of either Shih Tzu or Lhasa Apso, of both sex, that were, dewormed, vaccinated, antibiotic-free at least 6 months prior to data collection, free from any other concurrent oral disorders, and for females, out of the estrus period. Specific inclusion criteria were in the control group (without dental calculus) the dogs should not present any signs of dental calculus and; in the experimental group (with dental calculus) the dogs should necessarily present signs of dental calculus.

### Dental evaluation

Previously to collection of saliva samples, a visual clinical examination of the oral cavity was carried out in order to evaluate the dental conditions, concerning the presence or absence of dental calculus.

A visual oral inspection was carried out by a veterinary, where each participant of the study was categorized based on the criteria adopted to evaluate the dental calculus: 0 for absence of dental calculus; 1 for supragingival calculus covering not more than one-third of the exposed surface of the examined tooth; 2 for supragingival calculus covering more than one third but not more than two thirds of the exposed tooth surface and 3 for supragingival calculus covering more than two third of the exposed tooth surface [67, 68].

### Saliva collection and preparation

A non-invasive collection, without general anesthesia, and after 2 h of the animal fasting was performed. Whole saliva samples were collected under stimulation using Micro•SAL™ saliva collection device (Oasis Diagnostics® Corporation - Vancouver, WA, USA) for approximately 5 to 10 min. The tip end of the white absorbent collection pad of the device was inserted into the mouth of the dog where saliva pools and collect until the pad was saturated. Saliva was totally transferred from the absorbent part to the collection tube using the compression tube.

The samples were centrifuged at 14,000 g for 20 min at 4 °C and whole saliva supernatants (WSS) separated from the pellet. This was followed by the lyophilization of samples for further analyses. The total protein concentration of WSS was measured by Micro BCA Protein Assay Kit (Thermo Scientific Pierce, Rockford, IL, USA) with bovine serum albumin used as the standard and it was stored at − 80 °C until further analysis [69, 70].

### In–solution digestion

The equivalent of 20 μg of each WSS sample was dried by a rotary evaporator, denatured and reduced for 1 h at room temperatute by the addition of 50 μl of solution 1 (4 M urea, 10 mM dithiothreitol), and 50 mM NH4HCO3, pH 7.8. After, four-fold dilution with 50 mM NH4HCO3, pH 7.8, tryptic was carried out for 16 h at 37 °C, after the addition of 4% (w/w) sequencing-grade trypsin (Promega, USA). Finally, aliquots from each sample were dried again in a rotary evaporator, de-salted by C18 Pipette Tips (Millipore, USA) and finally subjected to mass spectrometry analyses [71].

### Mass spectrometry analyses

Mass spectrometry analyses were carried in triplicates for each sample with a nano-HPLC Proxeon (Thermo Scientific, San Jose, CA, USA) which allows in-line liquid chromatography with the capillary column, 60 μm × 100 mm (Pico Tip™ EMITTER, New Objective, Woburn, MA) filled with C^18^ resin of 5 mm diameter and 200Ǻ pores sizes (Michrom BioResources, Auburn, CA) linked to the mass spectrometer (LTQ-Velos, Thermo Scientific, San Jose, CA, USA) using an electrospray ionization in a survey scan in the range of m/z values 390–2000 tandem MS/MS.

The equivalent of 20 μg of each sample already dried by rotary evaporator was re-suspended in 20 μg of 0.1% formic acid and then subjected to reversed-phase LC-ESI-MS/MS. The nano-flow reversed-phase HPLC was developed with linear gradient of 85 min ranging from 0 to 100% of solvent B (97.5% acetonitrile, 0.1% formic acid) at a flow rate of 200 nl/min with a maximum pressure of 280 bar. Electrospray voltage and the temperature of the ion transfer capillary were 1.8 kV and 250 °C respectively.

### Database searches

The acquired MS/MS spectra were compared to canus lupus familiaris protein database (UniPROT and TREMBL, Swiss Institute of Bioinformatics, Geneva, Switzerland, http://ca.expasy.org) using SEQUEST and Proteome Discoverer 1.3 software (Thermo, USA). In order to infer protein with high confidence, the SEQUEST filter criteria applied to MS/MS spectra were: 1.5; 2.5; 3.1; 3.1; 4.5 for the XCorr applied in addition to the Percolator filter. Search results were filtered at a false discovery rate of 1% using a reverse database search strategy [26, 27].

As described previously [26], after identification of the proteome profile, the most abundant proteins had their biological functions verified through the accession number using the database www.uniprot.org. In addition, the STRING database (http://string-db.org/) was searched for protein-proteins networks in the group of dogs without dental calculus and the group with dental calculus separately.

### Statistical analyses

The results obtained were analysed by SPSS for WINDOWS, version 19.0 (SPSS Inc., Chicago, IL, USA), using descriptive statistics. Additionally, Student’s t-test was used to compare total protein concentrations between the groups with and without dental calculus and between genders. And, Pearson’s chi-square test was chosen to examine the null hypothesis that there is no relationship between animal age, saliva volume and total protein in the saliva. Significance levels were set at 5% (*p* < 0.05).

## Supplementary information

**Additional file 1: Supplementary Table S1.** Proteins common to all participating dogs. **Supplementary Table S2.** Specific proteins identified in dogs without dental calculus. **Supplementary Table S3.** Specific proteins identified in dogs with dental calculus. **Supplementary Table S4.** Peptide sequence of the most abundant proteins from all groups and; **Supplementary Table S5.** Proteins-protein relationship involved in Reactome pathways in the group of dogs with dental calculus.

**Additional file 2: Supplementary Figure 1.** Protein-protein interaction network of the specific proteins identified in dogs without dental calculus, based on STRING database and showing only connected proteins. Most abundant proteins in the network (Table 3) are marked with a rectangular outline. Legend: Amyloid-beta A4 protein (APP); Rho GDP dissociation inhibitor (GDI) alpha (ARHGDIA); Additional sex combs like transcriptional regulator 1 (ASXL1); BUB1 mitotic checkpoint serine/threonine kinase (BUB1); Calmodulin 3 (phosphorylase kinase, delta) (CALM3); Chemokine (C-C motif) receptor 7, belongs to the G-protein coupled receptor 1 family (CCR7); Centrosomal protein 152 kDa (CEP152); Centrosomal protein 162 kDa (CEP162); Chromodomain helicase DNA binding protein 3 (CHD3); Choroideremia (Rab escort protein 1) (CHM); Cytoplasmic linker associated protein 1 (CLASP1); CAP-GLY domain containing linker protein 1 (CLIP1); Component of oligomeric golgi complex 2 (COG2); Deltex 3 like, E3 ubiquitin ligase (DTX3L); Histone H2B (ENSCAFG00000031879); GTP binding protein (GTPBP2); Host cell factor C1 (HCFC1); HECT and RLD domain containing E3 ubiquitin protein ligase family member 1 (HERC1); Histidine-rich glycoprotein (HRG); Integrin beta (ITGB2); Uncharacterized protein, Kalirin, RhoGEF kinase (KALRN); Keratin, type I cytoskeletal 10 (KRT10); Keratin 24, belongs to the intermediate filament family (KRT24); Keratin 5, belongs to the intermediate filament family (KRT5); Keratin, type I cytoskeletal (KRT9); Histone H3 (LOC488263); Mitogen-activated protein kinase 6 (MAPK6); Neural precursor cell expressed, developmentally down-regulated 4-like, E3 ubiquitin protein ligase (NEDD4L); Neuromedin U receptor 1, belongs to the G-protein coupled receptor 1 family (NMUR1); Papillary renal cell carcinoma (translocation-associated) (PRCC); RAN binding protein 2 (RANBP2); Ras protein-specific guanine nucleotide-releasing factor 2 (RASGRF2); RAB6A GEF complex partner 1 (RIC1); RNA binding protein S1, serine-rich domain (RNPS1); Reticulon 4 (RTN4); Sphingosine-1-phosphate receptor 3, belongs to the G-protein coupled receptor 1 family (S1PR3); Serine/arginine repetitive matrix 2 (SRRM2); Serine/threonine kinase 10 (STK10); Transferrin (TF); Talin 2 (TLN2); Ubiquitin protein ligase E3 component n-recognin 1 (UBR1).Representation of protein-protein network inside the group of dogs without dental calculus with confidence score adopted at highest confidence-0.900

**Additional file 3: Supplementary Figure 2.** Protein-protein interaction network of the specific proteins identified in dogs with dental calculus, based on STRING database and showing only connected proteins. Most abundant proteins present in the network (Table 4) are marked with a rectangular outline. Legend: ATP-binding cassette, sub-family A, member 13 (ABCA13); Actinin, alpha 4 (ACTN4); AT hook containing transcription factor 1 (AHCTF1); Fructose-bisphosphate aldolase (ALDOA); Annexin (ANXA1); ATR serine/threonine kinase (ATR); B9 protein domain 2; *Canis lupus familiaris* transforming growth factor, beta 1 (TGFB1), mRNA (B9D2); *Canis lupus familiaris* calcium-sensing receptor (CASR); Centriolar coiled coil protein 110 kDa (CCP110); Cyclin-dependent kinase 12 (CDK12); *Canis lupus familiaris* carcinoembryonic antigen-related cell adhesion molecule 1 (CEACAM1); Uncharacterized protein (CENPF), Centrosomal protein 250 kDa (CEP250); Cytoplasmic linker associated protein 1 (CLASP1); Cytoplasmic linker associated protein 2 (CLASP2); Ceruloplasmin (ferroxidase); Belongs to the multicopper oxidase family (CP); Dedicator of cytokinesis 2; Belongs to the DOCK family (DOCK2); Cyclin N-terminal domain-containing protein (ENSCAFG00000016600); Fas (TNFRSF6) binding factor 1 (FBXO43); Fibronectin (FN1); Uncharacterized protein (GOLGA2); General transcription factor IIF, polypeptide 1, 74 kDa (GTF2F1); HECT domain containing E3 ubiquitin protein ligase 1 (HECTD1); Uncharacterized protein; Belongs to the heat shock protein 70 family (HSPA8); Heat shock protein beta-1 (HSPB1); HECT, UBA and WWE domain containing 1, E3 ubiquitin protein ligase (HUWE1); Histone-lysine N-methyltransferase; Lysine (K)-specific methyltransferase 2D (KMT2D); Keratin 13 (KRT13); Keratin 3 (KRT3); L-lactate dehydrogenase (LDHA); Glyceraldehyde-3-phosphate dehydrogenase (LOC477441); Histone H3 (LOC483167); Matrix metalloproteinase-9 (MMP9); Nibrin (NBN); non-SMC condensin II complex, subunit D3 (NCAPD3); Condensin complex subunit 2 (NCAPH); Nuclear receptor corepressor 1 (NCOR1); Nuclear receptor corepressor 2 (NOL10); Nucleolar protein 6 (NOL6); Profilin 1 (PFN1); Phosphatidylinositol binding clathrin assembly protein (PICALM); Polycystin 2 (PKD2); Serine/threonine-protein kinase PLK; Polo-like kinase 1 (PLK1); Polymerase (DNA directed), epsilon, catalytic subunit (POLE); Protein phosphatase 1, regulatory subunit 12A (PPP1R12A); Protein tyrosine kinase 2 (PTK2); Uncharacterized protein (RAB3IP); RB1-inducible coiled-coil 1 (RB1CC1); RB binding protein 6, ubiquitin ligase; Retinoblastoma binding protein 6 (RBBP6); Regulatory associated protein of MTOR, complex 1 (RPTOR); U4/U6.U5 tri-snRNP-associated protein 1; Squamous cell carcinoma antigen recognized by T cells (SART1); SEC31 homolog A, COPII coat complex component; SEC31 homolog A (SEC31A); SET domain containing 1A (SETD1A); SH2 domain containing adaptor protein B (SHB); Spectrin, beta, non-erythrocytic 1 (SPTBN1); Uncharacterized protein (SRGAP2); Transaldolase (TALDO1); Transcobalamin I (TCN1); Thrombospondin 1 (THBS1); Tenascin R (TNR); Tumor protein p53 binding protein 1 (TP53BP1); Trafficking protein particle complex 6A (TRAPPC6A); Thyroid hormone receptor interactor 12 (TRIP12); U-box domain containing 5 (UBOX5); Unc-51 like autophagy activating kinase 2 (ULK2); Versican (VCAN). Representation of protein-protein network inside the group of dogs with dental calculus with confidence score adopted at highest confidence-0.900.

## Data Availability

The datasets supporting the conclusion of this article are included within the article and Additional files.

## Abbreviations

AEP: Acquired enamel pellicle
PRPs: Proline-rich proteins
WSS: Whole saliva supernatants
Micro BCA: Micro Bicinchoninic Acid
S1P1: Sphingosine-1-Phosphate Receptor Protein 1
TNF: Tumor necrosis factor
MMPs: Matrixmetalloproteinases
TGFB1: Transforming growth factor beta
C8B: Complement C8 beta chain
CaSR: Calcium-sensing receptor
Ca^2+^: Calcium
TRPC3: Transient receptor potential canonical 3
ROCK: Rho kinase
