# Supplementary figures and images for: Salivary proteomic profile of dogs with and without dental calculus
Source: BMC Vet Res. 2020 Aug 19;16:298. doi: 10.1186/s12917-020-02514-0 (PMC7437026; doi:10.1186/s12917-020-02514-0)

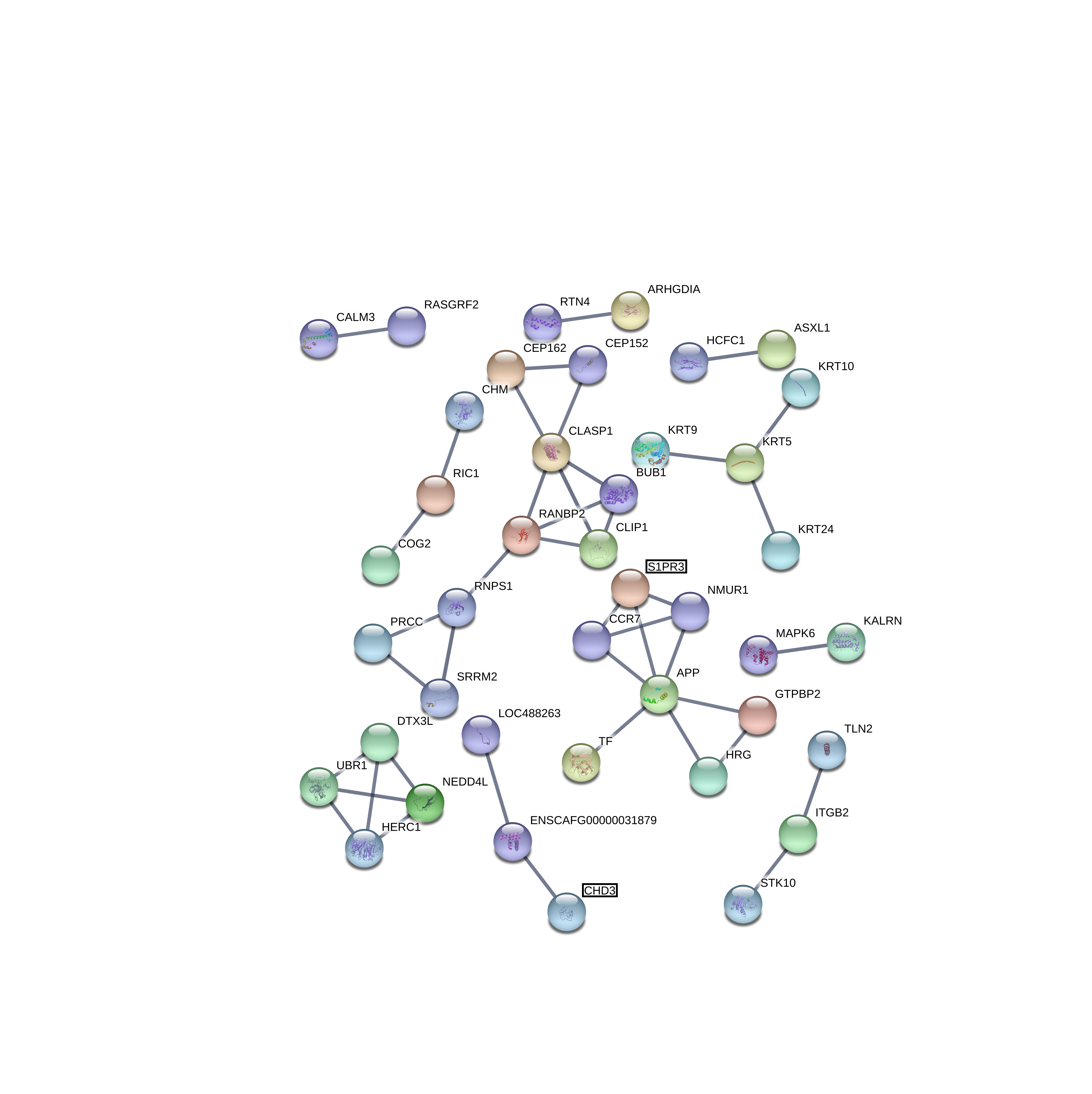

Supplement: Supplementary file 2 — Additional file 2: Supplementary Figure 1. Protein-protein interaction network of the specific proteins identified in dogs without dental calculus, based on STRING database and showing only connected proteins. Most abundant proteins in the network (Table 3) are marked with a rectangular outline. Legend: Amyloid-beta A4 protein (APP); Rho GDP dissociation inhibitor (GDI) alpha (ARHGDIA); Additional sex combs like transcriptional regulator 1 (ASXL1); BUB1 mitotic checkpoint serine/threonine kinase (BUB1); Calmodulin 3 (phosphorylase kinase, delta) (CALM3); Chemokine (C-C motif) receptor 7, belongs to the G-protein coupled receptor 1 family (CCR7); Centrosomal protein 152 kDa (CEP152); Centrosomal protein 162 kDa (CEP162); Chromodomain helicase DNA binding protein 3 (CHD3); Choroideremia (Rab escort protein 1) (CHM); Cytoplasmic linker associated protein 1 (CLASP1); CAP-GLY domain containing linker protein 1 (CLIP1); Component of oligomeric golgi complex 2 (COG2); Deltex 3 like, E3 ubiquitin ligase (DTX3L); Histone H2B (ENSCAFG00000031879); GTP binding protein (GTPBP2); Host cell factor C1 (HCFC1); HECT and RLD domain containing E3 ubiquitin protein ligase family member 1 (HERC1); Histidine-rich glycoprotein (HRG); Integrin beta (ITGB2); Uncharacterized protein, Kalirin, RhoGEF kinase (KALRN); Keratin, type I cytoskeletal 10 (KRT10); Keratin 24, belongs to the intermediate filament family (KRT24); Keratin 5, belongs to the intermediate filament family (KRT5); Keratin, type I cytoskeletal (KRT9); Histone H3 (LOC488263); Mitogen-activated protein kinase 6 (MAPK6); Neural precursor cell expressed, developmentally down-regulated 4-like, E3 ubiquitin protein ligase (NEDD4L); Neuromedin U receptor 1, belongs to the G-protein coupled receptor 1 family (NMUR1); Papillary renal cell carcinoma (translocation-associated) (PRCC); RAN binding protein 2 (RANBP2); Ras protein-specific guanine nucleotide-releasing factor 2 (RASGRF2); RAB6A GEF complex partner 1 (RIC1); RNA binding pro [file 12917_2020_2514_MOESM2_ESM.tif]

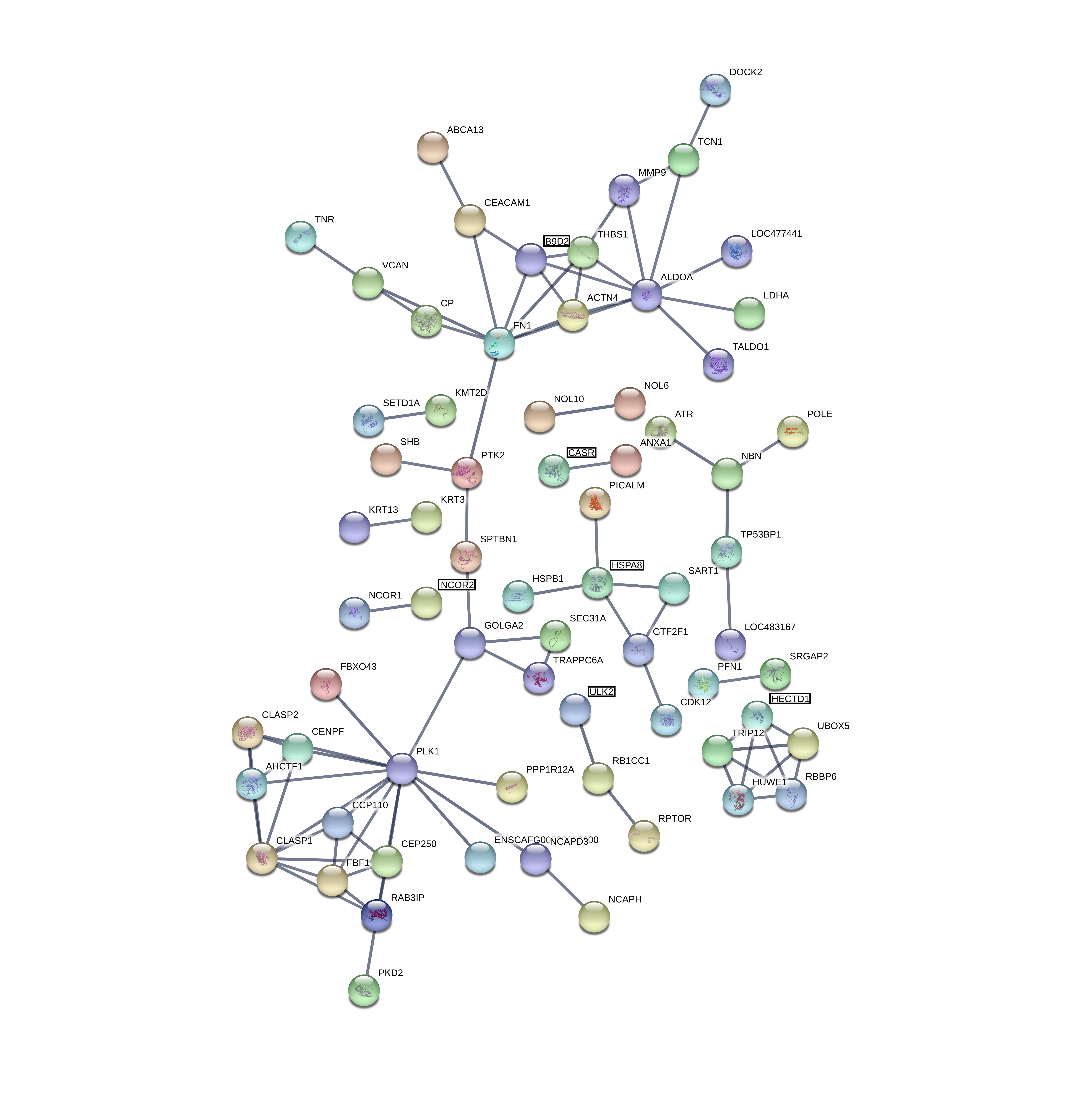

Supplement: Supplementary file 3 — Additional file 3: Supplementary Figure 2. Protein-protein interaction network of the specific proteins identified in dogs with dental calculus, based on STRING database and showing only connected proteins. Most abundant proteins present in the network (Table 4) are marked with a rectangular outline. Legend: ATP-binding cassette, sub-family A, member 13 (ABCA13); Actinin, alpha 4 (ACTN4); AT hook containing transcription factor 1 (AHCTF1); Fructose-bisphosphate aldolase (ALDOA); Annexin (ANXA1); ATR serine/threonine kinase (ATR); B9 protein domain 2; Canis lupus familiaris transforming growth factor, beta 1 (TGFB1), mRNA (B9D2); Canis lupus familiaris calcium-sensing receptor (CASR); Centriolar coiled coil protein 110 kDa (CCP110); Cyclin-dependent kinase 12 (CDK12); Canis lupus familiaris carcinoembryonic antigen-related cell adhesion molecule 1 (CEACAM1); Uncharacterized protein (CENPF), Centrosomal protein 250 kDa (CEP250); Cytoplasmic linker associated protein 1 (CLASP1); Cytoplasmic linker associated protein 2 (CLASP2); Ceruloplasmin (ferroxidase); Belongs to the multicopper oxidase family (CP); Dedicator of cytokinesis 2; Belongs to the DOCK family (DOCK2); Cyclin N-terminal domain-containing protein (ENSCAFG00000016600); Fas (TNFRSF6) binding factor 1 (FBXO43); Fibronectin (FN1); Uncharacterized protein (GOLGA2); General transcription factor IIF, polypeptide 1, 74 kDa (GTF2F1); HECT domain containing E3 ubiquitin protein ligase 1 (HECTD1); Uncharacterized protein; Belongs to the heat shock protein 70 family (HSPA8); Heat shock protein beta-1 (HSPB1); HECT, UBA and WWE domain containing 1, E3 ubiquitin protein ligase (HUWE1); Histone-lysine N-methyltransferase; Lysine (K)-specific methyltransferase 2D (KMT2D); Keratin 13 (KRT13); Keratin 3 (KRT3); L-lactate dehydrogenase (LDHA); Glyceraldehyde-3-phosphate dehydrogenase (LOC477441); Histone H3 (LOC483167); Matrix metalloproteinase-9 (MMP9); Nibrin (NBN); non-SMC condensin II complex, subunit D3 (NCAPD3); Conden [file 12917_2020_2514_MOESM3_ESM.tif]
